# Leptospirosis in Central Romania: A 17-Year Single-Center Cohort Study of Hospitalized Adults

**DOI:** 10.3390/microorganisms14020298

**Published:** 2026-01-27

**Authors:** Victoria Birlutiu, Rares-Mircea Birlutiu

**Affiliations:** 1 Faculty of Medicine, Lucian Blaga University of Sibiu, 550169 Sibiu, Romania; 2 County Clinical Emergency Hospital, 550245 Sibiu, Romania; 3 Department 14-Orthopedics, Anaesthesia Intensive Care Unit, Faculty of Medicine, “Carol Davila” University of Medicine and Pharmacy, 020021 Bucharest, Romania; 4 Foisor Clinical Hospital of Orthopedics, Traumatology, and Osteoarticular TB, 030167 Bucharest, Romania

**Keywords:** leptospirosis, clinical manifestations, Romania, treatment

## Abstract

Leptospirosis is an important zoonosis that can present as a self-limited influenza-like illness or progress to severe, including life-threatening multiorgan dysfunction. We report the epidemiology, clinical profile, and correlates of severity among adults hospitalized patients with leptospirosis diagnosed in central Romania over a period of 17 years. We conducted a retrospective, single-center cohort study of adults admitted between 1 January 2008 and 1 December 2025 with laboratory-confirmed leptospirosis. Confirmation was based on positive anti-*Leptospira* IgM serology, with repeat testing when the initial result was equivocal and confirmation with a microscopic agglutination test. We extracted demographic, exposure, clinical, laboratory, treatment, and outcome data from medical records. The modified Faine score was also calculated using admission data. Sixty-four patients were included in this analysis, of which 53 (82.8%) were male patients. Admissions peaked in 2023–2025 (34/64, 53.1%) and in the August–September months. Reported exposures were predominantly peri-domestic (46.9%), followed by rural/animal-related occupations (20.3%) and freshwater contact (17.2%). Severe disease occurred in 26/64 (40.6%), was more frequent in men (*p* = 0.021), and was more common pre-pandemic than during/after the pandemic (*p* < 0.001). Severe cases were associated with oliguria/anuria, hematuria, and jaundice, alongside higher urea/creatinine and bilirubin, lower hemoglobin and lymphocyte percentages, and a longer hospitalization period. One in-hospital death occurred (1.6%). Serogroup identification was available for 10 patients (15.6%) (pre-pandemic only). The mean modified Faine score was 27.5 ± 6.0. In this temperate-region cohort study, hospitalized leptospirosis showed a marked male predominance, a late-summer peak, and a substantial burden of severe disease. Early renal and hepatobiliary manifestations with concordant laboratory abnormalities may support timely risk stratification and escalation of care, while expanded molecular diagnostics and systematic typing are needed to clarify temporal trends and guide prevention.

## 1. Introduction

Leptospirosis is a major global public healthcare concern, accounting for approximately one million cases annually, with reported case-fatality rates ranging from 6.85% to 10% [1,2], and reaching up to 15% in severe forms of disease [3].

The causative pathogen is a spirochaete bacterium of the genus *Leptospira* (most commonly *Leptospira interrogans*), typically transmitted through exposure to urine from infected rodents, which contaminates stagnant freshwater. Human infection may occur during recreational or occupational activities, including swimming and fishing, as well as work in abattoirs, rice paddies, and sugarcane or banana plantations [4], though exposure may also occur during the consumption of non-potable water. Transmission takes place via breaches in the skin or through mucosal surfaces (conjunctival, inhalational/respiratory, oral, or genital), particularly during the warm season in temperate climates. The clinical spectrum ranges from asymptomatic infection and mild, influenza-like illness to severe disease complicated by acute hepatic failure, acute kidney injury, and meningoencephalic or cardiac involvement [5], including severe hemorrhagic syndromes [6].

Clinical severity deserves particular attention: patients with severe icterohemorrhagic disease are more likely to present and be correctly recognized, whereas mild or moderate presentations are inherently at risk of underdiagnosis or misclassification. From a therapeutic perspective, current practice remains consistent with guidance—aminopenicillins and doxycycline continue to be appropriate first-line options against *Leptospira* in most clinical scenarios [6].

At a global scale, climate change is reflected by a 1.09 °C increase in near-surface global temperature during the 2011–2020 decade, with a projected warming of 1.4–4.4 °C by 2100, together with the rising frequency of extreme weather events, particularly heavy rainfall and flooding, which are expected to increase the risk of waterborne infections, including leptospirosis [7]. Rising summer temperatures together with increasingly intense precipitation in recent years, as previously mentioned, also create favorable ecological conditions for zoonotic pathogens in our region. As with other zoonoses, the local epidemiology must be continuously reassessed, even if the topic is not new, because climate change, extreme weather events, recurrent heavy rainfall, and flooding collectively amplify the risk of leptospirosis outbreaks and are plausibly associated with more severe clinical courses.

The genus *Leptospira* has historically been described as comprising two principal species: the pathogenic *L. interrogans* and the saprophytic *L. biflexa*. Serologically, *Leptospira* are classified into serogroups and serovars; approximately 240 serovars have been recognized, although only a subset are pathogenic. From a genotypic perspective (DNA–DNA hybridization), 21 species have been identified, of which 9 are responsible for human infection, while an additional 5 are considered intermediately pathogenic [8].

Globally, most cases (approximately 73%) occur in tropical regions, Sub-Saharan Africa, Southeast Asia, Oceania, and the Caribbean, although leptospirosis also occurs in temperate settings, predominantly during the warm season [9,10,11]. At the European level, surveillance data underscore this changing landscape. Between 2010 and 2021, 12180leptospirosis cases were reported across 23 EU/EEA countries, 79% of which originated from five countries (France, Germany, The Netherlands, Portugal, and Romania) [9].

Transmission is primarily associated with contact of abraded skin or mucosal surfaces with water contaminated by urine, most commonly from rodents, which may shed leptospires for several months after infection; other wild and domestic animals can also act as reservoirs, excreting leptospires that persist within the renal tubules. Alternative transmission routes are exceptional, including person-to-person or sexual transmission [10], transmission during lactation via breast milk [11], and transplacental transmission [12].

We hypothesized that climate change may drive shifts in seasonality, circulating serovars, and/or the clinical spectrum of disease. Accordingly, we aimed to evaluate pre-pandemic versus post-pandemic leptospirosis cases managed at a single adult Infectious Diseases center located in central Romania.

## 2. Materials and Methods

### 2.1. Study Design and Setting

We conducted a retrospective, single-center cohort study of patients hospitalized with leptospirosis in Sibiu Clinical County Hospital, Romania from 1 January 2008 to 1 December 2025.

### 2.2. Participants and Eligibility

All consecutive inpatients aged 18 years old or above (N = 64) with a laboratory-confirmed diagnosis of leptospirosis were included in this analysis. Confirmation required a positive anti-Leptospira IgM ELISA, with repeat (paired) testing in cases in which the initial result was equivocal. Serologic confirmation was performed with the microscopic agglutination test (MAT), the reference standard for leptospirosis, which detects antibodies to a panel of live *Leptospira* serovars; confirmation was defined by a ≥4-fold rise between acute and convalescent sera or seroconversion, while a single high titer (≥1:200) was considered supportive when interpreted with the clinical–epidemiologic context. In the early years of this study, the broadly reactive Patoc antigen—the non-pathogenic *Leptospira biflexa* serovar Patoc 1—was used. Where available in the pre-pandemic era, additional laboratory evidence (e.g., PCR) was abstracted from the laboratory record; serogroup/species assignments were captured as reported by the diagnostic laboratory.

### 2.3. Data Source and Variables

Data were abstracted from the electronic medical record, the laboratory information system, discharge summaries, and a standardized epidemiological history form completed at admission. Variables included the following: Demographics and context: age, sex, residence (urban/rural), year and month of hospitalization, and pre-pandemic indicator. Clinical features/exposures: symptoms (e.g., fever, headache, myalgia, meningism), epidemiologic exposures (freshwater contact, fishing/bathing/fall into water, animal or farm contact, rural travel), and coinfections. Comorbidity/organ failure: hypertension, diabetes, COPD, chronic hepatitis, cardiovascular comorbidity, hematologic disease, chronic alcoholism, obesity, neoplasia, and acute renal failure (AKI) and acute hepatic injury (AHI) as recorded by clinicians. Laboratory tests at admission. Treatment and outcomes. A geospatial variable storing the patient locality was created for mapping; no personal identifiers were recorded. Both authors assessed the data retrieval and consistency.

### 2.4. Case Severity

Clinical severity was dichotomized as moderate vs. severe (icterohemorrhagic) according to treating-team documentation in the medical record.

### 2.5. Faine (Modified) Diagnostic Score

The World Health Organization has incorporated Faine’s criteria to improve the timeliness and yield of leptospirosis diagnosis, and it changed them in 2012, producing the modified Faine’s criteria [13,14]. For each case, we computed a Faine-style composite using admission data. Part A (clinical): headache (2), fever (2), fever > 39 °C (2), conjunctival suffusion (4), meningism (4), myalgia (4), bonus for concurrent suffusion + meningism + myalgia (+10), jaundice (1), albuminuria/nitrogen retention (2; counted if albuminuria present, BUN > 20 mg/dL, or creatinine > 1.2 mg/dL), and hemoptysis/dyspnea (2). Part B (epidemiology): rainfall (5), contact with contaminated environment (4), and animal contact (1). Part C (laboratory/microbiology): culture positive (25), PCR positive (25), ELISA IgM positive (15), SAT positive (15), other rapid tests (15), MAT single high titer (15), and MAT rising titer/seroconversion (25). Interpretation: Presumptive leptospirosis if A ≥ 26, A + B ≥ 26, or A + B + C ≥ 25; Possible if total 20–25. When a datum was unavailable, it was left missing (no imputation). For free-text exposures, we created transparent keyword rules (e.g., “river/lake/bathing/fishing/flood/animal contact”) to generate binary indicators.

### 2.6. Statistical Analysis

All analyses were two-sided with α = 0.05. Continuous variables were summarized by the mean (SD) and range; for markedly skewed distributions, we also inspected medians and IQRs. Categorical variables were reported as counts (percent). Group comparisons: continuous outcomes by severity were compared with the Mann–Whitney U (with Hodges–Lehmann shift and 95% CI when requested). Categorical variables were compared using Pearson’s χ^2^ or Fisher’s exact (two-sided) when expected counts were <5; linear-by-linear tests assessed ordinal trends. Software: analyses were performed in IBM SPSS Statistics version 29 software, and for maps, we used a Google Maps basemap in WGS-84 (World Geodetic System 1984).

### 2.7. Ethics

Written informed consent was obtained from all subjects involved in this study. Our study was conducted in accordance with the principles of the Declaration of Helsinki and was approved by the Institutional Ethics Committee (approval number 35112/23 December 2025).

## 3. Results

Across the 64 laboratory-confirmed leptospirosis cases hospitalized in our institution between 1 January 2008 and 1 December 2025, 53 (82.8%) were male patients. Serologic screening with the IgM enzyme-linked immunosorbent assay (IgM ELISA) was positive in all cases, supporting the clinical suspicion and contributing to laboratory confirmation. Among the 64 enrolled patients, 33 (51.6%) resided in urban areas and 31 (48.4%) in rural areas. Admissions were distributed across the entire study interval but were disproportionately concentrated in recent years, with more than half recorded in 2023–2025 (34/64, 53.1%), and 30/64 (46.9%) cases occurred in the pre–COVID-19 period. In-hospital outcomes were favorable overall, with 63/64 (98.4%) discharged alive and 1 in-hospital death (1.6%). Month of admission was available for 64/64 cases (100%) and showed a clear seasonal pattern, peaking in August (13/64, 20.31%) and September (12/64, 18.75%), consistent with a summer predominance (30/64, 36.87%). Regarding clinical severity, 38/64 (59.4%) cases were classified as moderate, while 26/64 (40.6%) presented with a severe (icterohemorrhagic) form of the disease.

In terms of the symptom onset-to-admission interval and length of stay, of the 64 patients with complete data for these variables, the time from symptom onset to hospitalization ranged from 1 to 21 days, with a mean of 5.76 ± 3.43 days. The length of hospital stay ranged from 5 to 31 days, with a mean duration of 10.63 ± 4.85 days.

**Figure 1:**
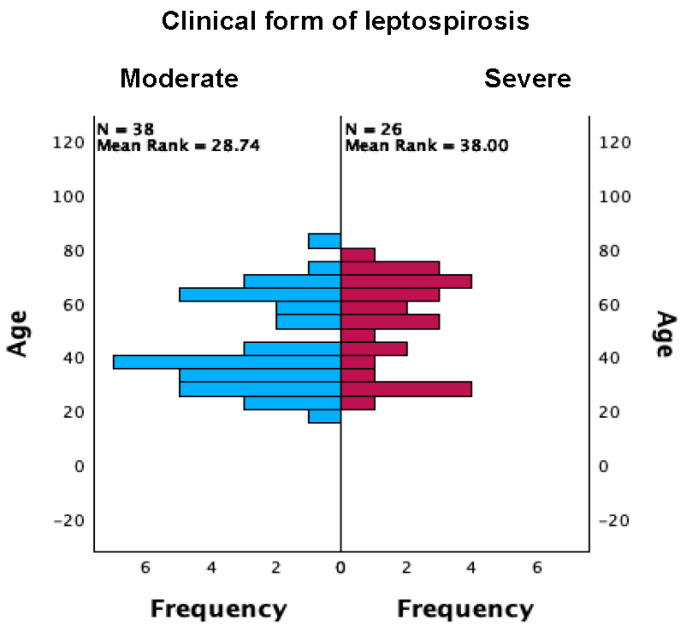
Age distribution by clinical form of leptospirosis.

The age distribution did not differ by survival status at discharge (Mann–Whitney U = 2.0, *p* = 0.094; asymptotic *p* = 0.110). The corresponding rank-biserial correlation is |r| ≈ 0.20, indicating a small, non-significant effect. The interpretation of the results is limited by the single death (1.6%). Age distributions differed and were borderline across severity categories on a non-parametric comparison (Mann–Whitney U = 637.0, two-sided *p* = 0.050, asymptotic), as shown in Figure 1. The associated standardized effect was r = Z/√N ≈ 0.25, indicating a small shift in age ranks.

In terms of organ involvement and comorbidities in our cohort of patients, they ranged from infrequent to moderate: acute hepatic injury (AHI) 5/64 (7.8%), acute kidney injury (AKI) 6/64 (9.4%), chronic hepatitis 6/64 (9.4%), other cardiovascular comorbidity 7/64 (10.9%), chronic obstructive pulmonary disease (COPD) 5/64 (7.8%), chronic alcohol use 8/64 (12.5%), obesity 16/64 (25.0%), hematologic disease 5/64 (7.8%), current smoking 2/64 (3.1%), hypertension 11/64 (17.2%), type-2 diabetes 3/64 (4.7%), and neoplasia 4/64 (6.3%). Most patients were discharged alive, and one in-hospital death occurred in our analysis, as was previously reported. Given the single unfavorable event, Fisher’s exact tests found no reliable associations between mortality and any predictor (demographics, calendar variables, clinical form, or comorbidities); results should be interpreted as sparse-event-limited rather than evidence of no effect.

Acute liver failure was strongly associated with a severe presentation and severe forms of the disease (6/6, 100% vs. 20/58, 34.5%; OR 24.41, 95% CI 1.31–455.40; RR 2.67, 95% CI 1.78–4.01; Fisher *p* = 0.003). Acute kidney injury showed a similar direction but was not statistically significant given the small population assessed in our cohort (4/5, 80.0% vs. 22/59, 37.3%; OR ≈ 6.73; Fisher *p* = 0.149).

A severe icterohemorrhagic presentation occurred in 26/64 (40.6%) cases. Severe disease was substantially more frequent in men than women (25/53 [47.2%] vs. 1/11 [9.1%]; OR 8.93, 95% CI 1.07–74.78; RR 5.19, 95% CI 0.78–34.36; Fisher *p* = 0.021). The pre-pandemic period carried a higher burden of severe cases than the during/after period (19/30 [63.3%] vs. 7/34 [20.6%]; OR 6.66, 95% CI 2.19–20.31; RR 3.08, 95% CI 1.51–6.28; Fisher *p* < 0.001). Acute hepatic injury was strongly associated with severity (6/6 [100%] vs. 20/58 [34.5%]; OR 24.41, 95% CI 1.31–455.40; RR 2.67, 95% CI 1.78–4.01; Fisher *p* = 0.003), as were other cardiovascular comorbidities (6/7 [85.7%] vs. 20/57 [35.1%]; OR 11.10, 95% CI 1.25–98.77; RR 2.44, 95% CI 1.53–3.89; Fisher *p* = 0.015). Directionally similar but underpowered signals were observed for AKI and COPD (each 4/5 [80.0%] vs. 22/59 [37.3%]; OR ≈ 6.73; Fisher *p* = 0.149 for both). No evidence of association was detected for the residence (rural 15/31 [48.4%] vs. urban 11/33 [33.3%]; OR 1.88, 95% CI 0.68–5.15; Fisher *p* = 0.309) or month of admission (*p* = 0.865). Year-wise heterogeneity reached significance (*p* = 0.012).

The distribution of symptom-onset-to-admission delay (1, 2, 3, 4, 5, 6, 7, 10, 14, 21 days) did not differ between moderate and severe icterohemorrhagic forms (Pearson χ^2^(9) = 13.21, *p* = 0.153; likelihood-ratio χ^2^(9) = 15.90, *p* = 0.069; linear-by-linear association *p* = 0.289). Rank correlations were small and non-significant (*p >* 0.25). Severe disease forms clustered for a longer hospitalization period (categories 10–31 days), whereas moderate cases concentrated at shorter hospitalization periods (5–11 days). The association was substantial and monotonic (Pearson χ^2^(13) = 19.31, *p* = 0.114; likelihood-ratio χ^2^(13) = 25.27, *p* = 0.021; linear-by-linear association *p* = 0.001; Pearson’s r = 0.490, Spearman’s ρ = 0.601, both *p* < 0.001). For clinical interpretability, a post hoc dichotomy at ≥10 days yielded 19/26 (73.1%) long stays in severe cases versus 3/20 (15.0%) in moderate cases, corresponding to OR 15.4 (95% CI 3.4–68.9) and RR 4.87 (95% CI 1.67–14.2). These effects support a markedly prolonged hospitalization among severe presentations of the disease in our cohort of patients.

### 3.1. Clinical Manifestations at the Time of Admission

At the time of presentation in our department, in terms of the clinical manifestations, the following symptoms predominated: fever occurred in 55/64 (85.9%) and chills in 50/64 (78.1%), accompanied by fatigue/asthenia (45/64, 70.3%), headache (34/64, 53.1%), and myalgia (33/64, 51.6%). Gastrointestinal involvement was also frequent, vomiting (25/64, 39.1%), loss of appetite (24/64, 37.5%), abdominal pain (21/64, 32.8%), nausea (20/64, 31.3%), and diarrhea (9/64, 14.1%). Renal and hepatobiliary findings were notable, with oliguria/anuria (23/64, 35.9%), hematuria (16/64, 25.0%), and jaundice (17/64, 26.6%). Respiratory and other manifestations were less common: cough (16/64, 25.0%), chest pain (5/64, 7.8%), dysphagia/odynophagia (5/64, 7.8%), and rash (5/64, 7.8%).

A crosstabulation between symptoms and disease severity (moderate vs. severe-icterohemorrhagic) was also performed. Symptom–severity patterns were dominated by renal and hepatobiliary signs. Oliguria/anuria and hematuria showed the strongest associations with severe disease (oliguria/anuria present in 21/26 [80.8%] severe vs. 2/38 [5.3%] moderate; OR_sev_ 75.6, 95% CI 13.46–424.68; Fisher *p* < 0.001; hematuria 13/26 [50.0%] vs. 3/38 [7.9%]; OR_sev_ 11.67, 95% CI 2.86–47.67; *p* < 0.001). Jaundice was likewise enriched in severe presentations (14/26 [53.8%] vs. 3/38 [7.9%]; OR_sev_ 13.61, 95% CI 3.33–55.68; *p* < 0.001). Other symptoms were less discriminating: with loss of appetite (14/26 [53.8%] vs. 10/38 [26.3%]; OR_sev_ 3.27, 95% CI 1.14–9.39; Fisher *p* = 0.036) and fatigue (22/26 [84.6%] vs. 23/38 [60.5%]; OR_sev_ 3.59, 95% CI 1.03–12.50; Fisher two-sided *p* = 0.052) suggested a higher burden among severe cases, whereas fever, chills, headache, arthralgia, myalgia, dysphagia, cough, chest pain, diarrhea, and rash showed no statistically reliable differences (two-sided Fisher *p* ≥ 0.08). Rank correlations mirrored these findings: jaundice (Pearson’s *r* = 0.51, *p* < 0.001), oliguria/anuria (*r* = 0.77, *p* < 0.001), and hematuria (*r* = 0.48, *p* < 0.001) exhibited the largest monotonic associations with severity, whereas other symptoms had small, non-significant coefficients. Table 1 highlights our results.

Also, a crosstabulation between LOS and symptoms was assessed. Across 14 LOS categories (5–31 days), most symptom–LOS tables were sparse and not significant. Two symptoms demonstrated positive, monotonic relationships with a longer hospitalization: abdominal pain (Pearson’s *r* = 0.30, *p* = 0.040; Spearman’s ρ = 0.35, *p* = 0.016) and nausea (*r* = 0.35, *p* = 0.019). Other symptoms, including fever and chills, did not show consistent associations with the LOS (all *p >* 0.10 in trend tests).

### 3.2. Laboratory Test Results Performed at the Time of the Admission

Our cohort exhibited marked biochemical heterogeneity. Table 2 highlights the results of the performed laboratory studies. Renal indices were elevated on average, with a blood urea nitrogen (BUN) mean 80.9 mg/dL (95% CI 60.7–101.2) and creatinine 2.25 mg/dL (1.69–2.81). Hepatobiliary markers demonstrated a wide dispersion: total bilirubin 4.79 mg/dL (2.26–7.32), direct bilirubin 5.72 mg/dL (3.41–8.04), AST 128.7 U/L (84.4–173.0), ALT 161.9 U/L (84.1–239.6), GGT 193.2 U/L (147.4–239.0), ALP 164.0 U/L (142.9–185.1), and cholinesterase 4121.6 U/L (3792.7–4449.5). Coagulation parameters showed a modest prolongation on average (INR 1.161 [1.103–1.219]; aPTT 34.07 s [31.55–36.59]), with D-dimer elevation at 2698.6 mg/L (2127–3270). Tissue-injury enzymes were frequently raised (CK 341.5 U/L [230.1–452.8]; LDH 309.0 U/L [289.5–328.5]). Hematology-performed assays revealed WBCs 18.89 × 10^3^/µL (95% CI 3.26–34.52; range 3.91–50), hemoglobin 13.54 g/dL (13.13–13.95), hematocrit 38.94% (37.72–40.17), differential counts (neutrophils 73.49% [69.88–77.09], lymphocytes 16.05% [13.12–18.98], eosinophils 1.72% [1.31–2.12]), and platelets 150.9 × 10^3^/µL (123.0–178.8). Inflammatory markers were elevated on average (ESR 43.6 mm/h [36.2–51.1]; CRP 137.4 mg/L [112.8–161.9]), and fibrinogen averaged 534.5 g/L (491.5–577.5).

Using severe-minus-moderate contrasts with bootstrap 95% CIs, renal markers were higher in severe forms of the disease: BUN (Δ = +155.5; 95% CI 48.0–263.0) and creatinine (Δ = +2.99; 0.60–5.37). The cholestatic/hemolytic burden also appeared greater in severe cases: total bilirubin (Δ = +25.79; 1.20–50.38), direct (conjugated) bilirubin (Δ = +17.36; 0.56–34.16), and AST (Δ = +33.0; 3.0–63.0) showed non-overlapping CIs with zero. In contrast, ALT, γ-glutamyltransferase (GGT), alkaline phosphatase, cholinesterase, INR, and aPTT did not show a precise separation between groups. Regarding inflammatory/hematologic indices, ESR was higher in severe disease (Δ = +36; 12–60), whereas hemoglobin (Δ = −2.20; −3.00 to −1.40) and the lymphocyte percentage (Δ = −3.05; −4.10 to −2.00) were lower. The neutrophil percentage and platelets had wide CIs that crossed 0. C-reactive protein and fibrinogen were also imprecise. These patterns align with acute kidney and hepatobiliary injury, systemic inflammation, and relative lymphopenia in severe forms of leptospirosis. The effect-size output (Cohen’s d with very wide CIs) was generally consistent with the direction of these differences (e.g., d ≈ 1.02 for BUN; ≈0.89 for creatinine; ≈0.74 for total bilirubin; ≈0.78 for AST), but intervals were broad and frequently included 0, reflecting the tiny number of enrolled patients, and should not be over-interpreted.

### 3.3. Faine’s Criteria

Faine’s criteria for diagnosing leptospirosis form a scoring system using clinical (A), epidemiological (B), and lab (C) data, with a score of ≥26 in Part A or Part A + B, or ≥25 in Part A + B + C indicating presumptive leptospirosis, as was also calculated for our cohort of patients. Across the 64 cases assessed in our study, the Faine score averaged 27.47 ± 5.98 (range 18–46; 95% CI 26.02–28.95).

Faine scores tended to be higher in severe icterohemorrhagic forms of leptospirosis than in moderate cases (Mann–Whitney U = 354.5, Z = −1.919, two-sided *p* = 0.055; rank-biserial r ≈ 0.24, common-language effect ≈ 0.64 favoring severe). Faine scores did not differ by AHI status (Mann–Whitney U = 160.0, Z = −0.325, two-sided *p* = 0.746; exact *p* = 0.762), with a negligible effect (r ≈ 0.04; AUC ≈ 0.54). Faine scores did not differ by AKI status (Mann–Whitney U = 117.5, Z = −0.755, two-sided *p* = 0.450; exact *p* = 0.464; r ≈ 0.09), a null that likely reflects limited power (AKI *n* = 5).

### 3.4. Epidemiological Exposures

In our cohort of 64 leptospirosis cases, peri-domestic activities (gardening/household/wood handling) were most frequent (30/64; 46.9%), followed by rural/animal-related occupations (13/64; 20.3%) and freshwater contact (11/64; 17.2%; frequent fishing, accidental immersion, or lake bathing). Use of untreated well or spring water was reported by 5/64 (7.8%), rural travel/excursions by 3/64 (4.7%), and animal/food exposures by 2/64 (3.1%). These patterns underscore peridomestic and freshwater contexts as the principal risk environments in this setting.

### 3.5. Geographic Distribution of Leptospirosis (2008–2025)

**Figure 2:**
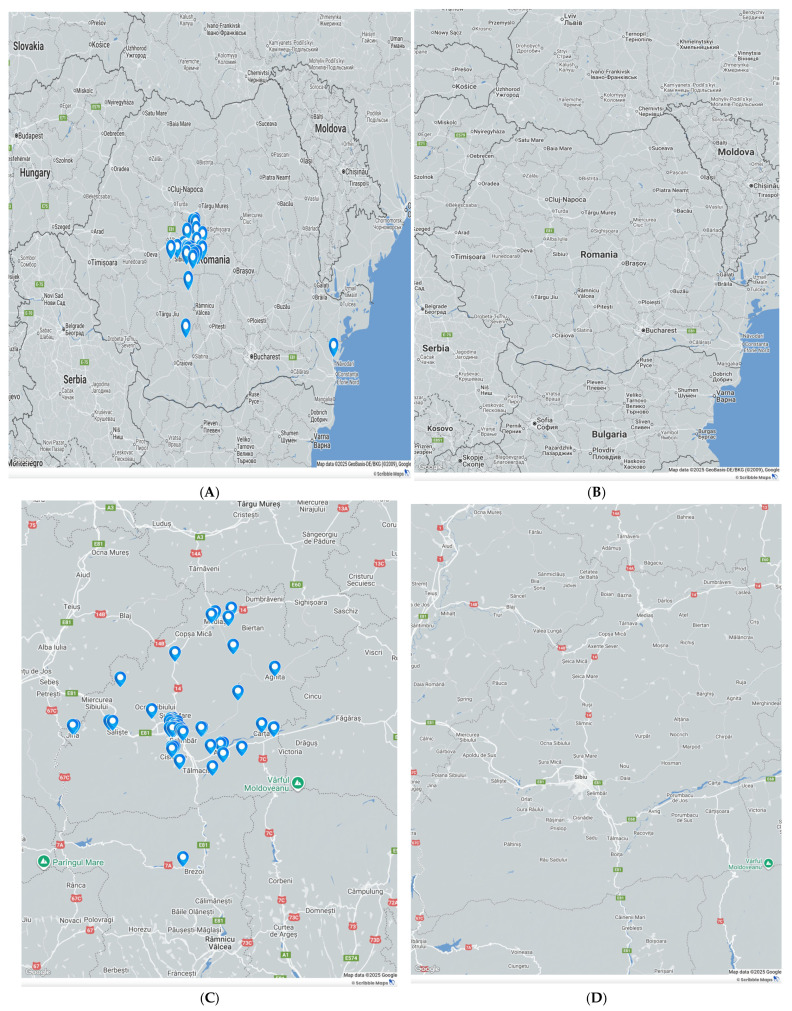
Geographic distribution of leptospirosis cases in Romania (2008–2025). Each map pin denotes one case (one patient) from the study cohort. Panel (**A**) shows the national overview; Panel (**C**) magnifies the cases diagnosed in our institution; Panels (**B**,**D**) provide the corresponding reference basemaps at matched scales. Coordinates are plotted in WGS-84 on a Google Maps basemap. Pins may overlap where multiple cases arose from the same locality. No personal identifiers are displayed.

To contextualize spatial patterns, we mapped the geographic distribution of confirmed leptospirosis cases in our department during the study period (2008–2025). Each pin represents one patient from the study cohort geocoded to the locality recorded in the study database; overlapping pins indicate multiple cases from the same area. The map highlights areas of higher case density while preserving anonymity (no personal identifiers displayed). Serogroup assignment was available in 10/64 (15.6%) cases—exclusively from the pre-pandemic testing era—most commonly Australis (50%) and Icterohaemorrhagiae (40%), with one mixed Pomona/Grippotyphosa identification (10%) (see Figure 2).

### 3.6. Treatment

In terms of the treatment, antibiotic therapy was predominantly with β-lactam-based drugs: ampicillin in 43 cases (21 moderate, 22 severe), ceftriaxone in 8 (4, 4), ceftazidime in 3 (1, 2), and amoxicillin/clavulanate in 5 (2, 3); doxycycline was used in 3 (2, 1), ciprofloxacin in 1 (1, 0), and moxifloxacin in 1 (1, 0). Coinfections were documented in 7 patients overall—Enterobacter spp. UTI 2 (0 moderate, 2 severe), Proteus spp. UTI 1 (1, 0), Candida auris-positive blood culture 1 (0, 1), and sepsis with MSSA 1 (severe), yielding 6/7 events among severe cases.

## 4. Discussion

The clinical presentation of leptospirosis remains heterogeneous, ranging from asymptomatic infection to severe disease with multiorgan failure. This variability is shaped by epidemiological determinants (including sanitation, socioeconomic conditions, and rodent infestation), host-related factors (individual susceptibility and occupational or recreational exposure), older age (particularly >60 years), and pathogen virulence characteristics, such as motility, route of entry, and the capacity to evade or withstand host defense mechanisms [15].

High-grade bacteremia has been associated with impaired recognition of *Leptospira* lipopolysaccharide by Toll-like receptor 4 (TLR4). Severe disease appears to be driven by a cytokine storm characterized by markedly elevated levels of interleukin (IL)-6, IL-10, and tumor necrosis factor (TNF)-α [16]. From a genetic susceptibility standpoint, individuals carrying the HLA-DQ6 alleles appear to have an increased risk of leptospiral infection [15,16]. Post-leptospirosis sequelae have been described, including nonspecific systemic symptoms, such as asthenia, myalgias, malaise, and persistent fatigue, as well as potential neurological and renal complications [17,18,19,20].

In our retrospective single-center cohort study of 64 laboratory-confirmed cases hospitalized between 2008 and 2025, leptospirosis predominantly affected men (82.8%), and admissions were disproportionately concentrated in 2023–2025 (53.1). Seasonality was pronounced, with a peak in August and September, consistent with the warm-season predominance in temperate climates and supporting the plausibility that environmental conditions and water exposure contribute substantially to risk in this setting. Epidemiological assessments indicated that peri-domestic activities (46.9%) represented the most frequent exposure context, followed by rural/animal-related occupations (20.3%) and freshwater contact (17.2%); use of untreated well/spring water was reported in a minority (7.8%). Severe clinical forms of leptospirosis accounted for 40.6% of admissions. Oliguria/anuria, hematuria, and jaundice showed the clearest associations with a severe presentation. Laboratory profiles further supported this clinical pattern. Severe disease was characterized by higher renal indices (BUN and creatinine) and a greater cholestatic/hemolytic burden (higher total and direct bilirubin), as well as higher AST; ESR was also higher, while hemoglobin and the lymphocyte percentage were lower in severe disease.

Following a pandemic-related dip in 2020, case counts increased during 2021–2022, with the incidence peaking in July–November and particularly notable rises in the Azores, mainland Portugal, and Madeira. According to the ECDC, 751 cases were recorded in 2022, 69% among adults aged 25–64 years; cases were predominantly male (male-to-female ratio ≈ 3:1), with a male peak at ages 45–64 (consistent with agricultural, wastewater, and other rodent-exposed occupations) and a female peak at ages 15–24, plausibly reflecting distinct exposure profiles [21]. These regional trends, together with our local observations, justify sustained clinical vigilance and targeted prevention strategies in high-risk settings.

*Leptospira* persists across the landscape in both rural and urban settings. In urban areas, infection rates are more often linked to recreational than occupational exposure and are associated with rodent infestation that contaminates soil, as well as infections among companion animals (in our series, two infections documented in dogs) [22,23,24,25,26,27].

In rural environments, the risk of contact with multiple potentially contaminated environmental sources is higher, consistent with the rural cases we report, where occupational exposures predominated over recreational ones (e.g., soil cultivation, animal husbandry, contact with contaminated stormwater, faulty sewage systems) [28,29].

With respect to Romania, relatively few studies are available—and these are largely from the veterinary domain, but water exposure (lakes, ponds, or flooded areas) is generally recognized as the most frequent route of contamination [30]. In the North-East region (2013–2015), 17 human cases were described, mostly men with a median age of 45 years, predominantly from rural settings; 82.35% were identified between August and November each year. Clinically, fever, malaise, asthenia, anorexia, and jaundice were common; hemorrhagic manifestations occurred in 40.05%. Serovars were confirmed as *L. icterohaemorrhagiae* in 8 patients, *L. pomona* in 1, and *L. wolffii* in 2 [31]. In Bucharest (2004–2014), a cohort of 132 patients, again mostly male, with a median age of 37 years, showed seasonality (68% May–September), with 51% from urban areas; 63% had respiratory manifestations, and 36% had coagulation abnormalities [32].

For hospitalized patients with leptospirosis, management includes supportive care with fluid and electrolyte repletion and acid–base correction. In cases complicated by acute kidney injury, efforts to maintain the urine output may include loop diuretics, and with renal replacement therapy, both hemodialysis and peritoneal dialysis can be implemented when clinically indicated [33], in addition to pathogen-directed (etiologic) therapy. For mild to moderate disease, doxycycline is generally regarded as an appropriate first-line option when initiated within the first 6 days of symptom onset [34]. Alternatively, azithromycin may be used in patients who do not tolerate doxycycline or penicillin [15]. For severe disease, recommended regimens include penicillin G, ampicillin, tetracyclines, ceftriaxone, or cefotaxime, selected and individualized according to the clinical severity, organ involvement, and patient-specific factors. From a therapeutic point of view, in our study, most patients received β-lactam-based regimens (predominantly ampicillin), with ceftriaxone and other agents used less commonly; doxycycline was rarely employed among hospitalized cases in this cohort. Coinfections were documented in a small subset and clustered largely among severe cases, emphasizing the importance of systematic evaluation for concomitant infections, and antimicrobial tailoring when clinical trajectories are atypical.

Comparable clinical patterns are reported elsewhere. In Bangladesh, >70% of patients had myalgia and jaundice, with anorexia and headache in >56% [35]. In India, serologic testing for leptospirosis confirmed 27–28% of acute febrile illnesses in tertiary-care settings [36,37], compared with 8.4% in Malaysia (Rafizah et al., 2013) and 7.5% in Thailand [38].

We also applied a Faine score using admission data. The cohort mean score was within the “presumptive leptospirosis” range, and scores tended to be higher in severe forms, although the difference was borderline. In practice, this supports the utility of structured clinical–epidemiological scoring systems to facilitate earlier recognition in resource-limited contexts.

Specific prophylaxis primarily focuses on the vaccination of companion animals; in a limited number of countries, human vaccination is also implemented, typically targeting serovars identified in local rodent reservoirs. Broader preventive strategies include public education regarding accidental and occupational exposure risks in endemic or high-risk settings, facilitating timely access to medical care, and ensuring a rapid diagnosis with a prompt initiation of pathogen-directed antimicrobial therapy [39].

Collectively, our findings underscore that, in central Romania, the infection risk may frequently arise from everyday peri-domestic behaviors and intermittent freshwater exposures.

Our study also has a few limitations that need discussion. Since this was a retrospective analysis, it may not offer a complete understanding of the clinical setting in the region at this time. Furthermore, as this is a single-center retrospective study, it may provide some heterogeneity when it comes to including the data. Furthermore, the number of enrolled patients is relatively small, and it does not represent a diverse range of hospitals nationwide but rather from our county and the surrounding counties. Finally, we only analyzed patients from a specific period; therefore, there is a possibility of residual confounding in our methodology. Our findings might not be nationally generalizable and probably should be interpreted as region-specific. Regardless of the previously mentioned limitations, this study also has some strengths. Our database used in this study was comprehensive and included a wide range of clinical and analytical variables at the time of hospital admission and during hospitalization.

## 5. Conclusions

In this retrospective, single-center adult cohort study from central Romania (2008–2025), leptospirosis remained a clinically relevant cause of hospitalization, characterized by a marked male predominance and a clear warm-season peak. A substantial proportion of patients presented with severe disease forms. A severe presentation was consistently linked to renal and hepatobiliary involvement, mainly oliguria/anuria, hematuria, and jaundice, and it was also supported by a specific laboratory profile (higher urea/creatinine, higher total/direct bilirubin, higher AST, increased inflammatory markers, and relative anemia/lymphopenia). Severe cases have also been associated with longer hospitalization periods, reinforcing the importance of early risk stratification at admission. Although hospitalizations were increasingly concentrated in recent years, serogroup/serovar characterization was available only pre-pandemic, limiting the potential for inference regarding temporal shifts in circulating strains or their relationship with the clinical severity. Prospective multicenter surveillance is needed to better define the evolving epidemiology, optimize early recognition, and support targeted prevention strategies in the context of changing environmental risks.

## Figures and Tables

**Table 1:** Symptoms by clinical severity of the disease.

Symptom	Moderate n/N (%)	Severe n/N (%)	OR for Severe (95% CI)	Fisher *p* (Two-Sided)
Fever	34/38 (89.5%)	21/26 (80.8%)	0.49 (0.12–2.05)	0.467
Chills (rigors)	31/38 (81.6%)	19/26 (73.1%)	0.61 (0.19–2.02)	0.541
Headache	20/38 (52.6%)	14/26 (53.8%)	1.05 (0.39–2.85)	1.000
Arthralgia	11/38 (28.9%)	10/26 (38.5%)	1.53 (0.53–4.41)	0.588
Myalgia	16/38 (42.1%)	17/26 (65.4%)	2.60 (0.92–7.30)	0.080
Fatigue/weakness	23/38 (60.5%)	22/26 (84.6%)	3.59 (1.03–12.50)	0.052
Abdominal pain	9/38 (23.7%)	12/26 (46.2%)	2.76 (0.94–8.09)	0.103
Nausea	11/38 (28.9%)	9/26 (34.6%)	1.30 (0.45–3.79)	0.784
Vomiting	13/38 (34.2%)	12/26 (46.2%)	1.65 (0.59–4.58)	0.436
Diarrhea	3/38 (7.9%)	6/26 (23.1%)	3.50 (0.79–15.54)	0.142
Loss of appetite	10/38 (26.3%)	14/26 (53.8%)	3.27 (1.14–9.39)	0.036
Dysphagia/Odynophagia	2/38 (5.3%)	3/26 (11.5%)	2.35 (0.36–15.14)	0.389
Cough	10/38 (26.3%)	6/26 (23.1%)	0.84 (0.26–2.69)	1.000
Chest pain	2/38 (5.3%)	3/26 (11.5%)	2.35 (0.36–15.14)	0.389
Jaundice	3/38 (7.9%)	14/26 (53.8%)	13.61 (3.33–55.68)	<0.001
Oliguria/Anuria	2/38 (5.3%)	21/26 (80.8%)	75.60 (13.46–424.68)	<0.001
Hematuria	3/38 (7.9%)	13/26 (50.0%)	11.67 (2.86–47.67)	<0.001
Rash	3/38 (7.9%)	2/26 (7.7%)	0.97 (0.15–6.27)	1.000

**Table 2:** Laboratory test results.

Laboratory Parameter	Minimum	Maximum	Mean	Std. Deviation
Blood urea nitrogen (mg/dL)	11.00	312.00	80.91	80.82
Creatinine (mg/dL)	0.59	10.06	2.24	2.22
Total bilirubin (mg/dL)	0.24	51.48	4.79	10.10
Direct (conjugated) bilirubin (mg/dL)	0.10	34.50	5.72	9.25
Aspartate aminotransferase (U/L)	10.00	1308.00	128.66	177.08
Alanine aminotransferase (U/L)	8.00	2441.00	161.85	311.53
Gamma glutamyltransferase (U/L)	10.00	666.00	193.18	182.00
Alkaline phosphatase (U/L)	54.00	345.00	164.00	84.79
Cholinesterase (U/L)	1826.00	6235.00	4121.58	1323.844
International normalized ratio	0.79	2.03	1.16	0.23
Activated partial thromboplastin time (s)	16.70	72.10	34.06	10.05
D-dimer (mg/dL)	327.00	6344.50	2698.62	2296.39
Creatine kinase (U/L)	9.00	2016.00	341.45	446.25
Lactate dehydrogenase (U/L)	169.00	456.00	309.00	78.07
White blood cells (10^3^ µL)	3.91	50.00	18.88	62.86
Hemoglobin (mg/dL)	9.60	16.50	13.54	1.60
Hematocrit (%)	26.90	48.40	38.94	4.90
Neutrophils (%)	41.60	93.90	73.48	14.41
Lymphocytes (%)	0.99	42.80	16.04	11.60
Eosinophils (%)	0.01	6.00	1.7159	1.656
Platelets (10^3^ µL)	18.00	518.00	150.88	111.77
Erythrocyte sedimentation rate (mm/h)	5.00	112.00	43.64	29.88
CRP (mg/dL)	4.26	373.60	137.35	99.60
Fibrinogen (mg/dL)	230.02	975.00	534.51	172.52

## Data Availability

The original contributions presented in this study are included in the article. Further inquiries can be directed to the corresponding authors.

## Abbreviations

The following abbreviations are used in this manuscript:

AHI	Acute hepatic failure
AKI	Acute kidney injury
ALP	Alkaline phosphatase
ALT	Alanine aminotransferase
AST	Aspartate aminotransferase
AUC	Area under the curve
BUN	Blood urea nitrogen
CI	Confidence interval
CK	Creatine kinase
COPD	Chronic obstructive pulmonary disease
COVID-19	Coronavirus disease 2019
CRP	C-reactive protein
DNA	Deoxyribonucleic acid
ELISA	Enzyme-linked immunosorbent assay
ESR	Erythrocyte sedimentation rate
GGT	Gamma-glutamyl transferase
HLA-DQ6	Human leukocyte antigen DQ6
IL	Interleukin
INR	International normalized ratio
IQR	Interquartile range
IgM	Immunoglobulin M
LDH	Lactate dehydrogenase
LOS	Length of stay
MAT	Microscopic agglutination test
MSSA	Methicillin-susceptible *Staphylococcus aureus*
OR	Odds ratio
PCR	Polymerase chain reaction
RR	Relative risk
SAT	Serum agglutination test
SD	Standard deviation
TLR4	Toll-like receptor 4
TNF-α	Tumor necrosis factor alpha
UTI	Urinary tract infection
WBC	White blood cell count
WGS-84	World Geodetic System 1984
